# New Approach for Detection of Dental Abnormalities: Observation of Teeth During Peroral Endoscopy

**DOI:** 10.7759/cureus.96158

**Published:** 2025-11-05

**Authors:** Ryo Kato, Kenta Hamada, Masaya Iwamuro

**Affiliations:** 1 Department of Gastroenterology and Hepatology, Okayama University Graduate School of Medicine, Dentistry, and Pharmaceutical Sciences, Okayama, JPN; 2 Department of Gastroenterology, Okayama Rosai Hospital, Okayama, JPN; 3 Department of Practical Gastrointestinal Endoscopy, Okayama University, Okayama, JPN

**Keywords:** endoscopy, periodontal disease (pd), teeth, teeth abnormalities, teeth caries

## Abstract

Upper gastrointestinal endoscopy, renowned for detecting esophageal, gastric, and duodenal lesions, now extends to spotting oral and pharyngeal abnormalities such as cancer. This study examined whether peroral endoscopy can be used to identify dental issues. Between July and September 2022, 72 patients had their teeth, predominantly molars, captured during peroral endoscopy. Dental abnormalities were found in 28 (39%) (95%CI, 27.7%-50.3%) cases, including pigmentation, residual plaque, root exposure, black spots suspected of caries, defects, and residual roots. Although limited, this study suggests that peroral endoscopy has the potential to detect dental problems, aid in timely treatment, and reduce risks associated with dental diseases.

## Introduction

The number of teeth has been linked to the incidence of dementia, falls, and reduced activities of daily living, possibly through its influence on oral hygiene, nutritional status, and systemic diseases [1-3]. Therefore, it is important to maintain oral health and avoid tooth extractions. In Japan, periodontal disease is the most common cause of tooth extraction (32.1%), followed by caries (29.2%) [4]. Periodontal disease is a condition wherein bacteria invading the periodontal pockets cause gingivitis or periodontitis. Normal periodontal pockets are less than 2 mm, but bacterial counts are known to increase at 4 mm or more. It has been reported that the majority of patients over 65 years of age have periodontal pockets of 4 mm or more [5]. In addition, 30% of patients over 20 years of age have untreated caries, and 40% of patients over 40 years of age had teeth extracted due to caries [6,7]. This situation is thought to be partly due to the low awareness of the concept of preventive dentistry in Japan. Compared with Sweden, a leading country in dental care, the rate of regular dental visits in Japan has been about 60% [5,8].

Upper gastrointestinal endoscopy has proven invaluable for evaluating lesions in the esophagus, stomach, and duodenum. This procedure, renowned for its high visibility, contributes significantly to the early detection and diagnosis of gastrointestinal diseases. In recent years, the utility of upper gastrointestinal endoscopy has expanded to include the detection of lesions beyond the upper gastrointestinal tract, such as pharyngeal and oral diseases, such as cancer [9,10]. Similarly, the possibility of utilizing endoscopic examinations passing through the oral cavity to detect dental abnormalities has been contemplated [11]. However, to the best of our knowledge, no study has examined the feasibility of detecting dental abnormalities during endoscopic examinations.

We considered that for patients who do not regularly visit dental clinics but undergo oral endoscopy, this examination could serve as an opportunity for simple dental screening and potentially lead to improved detection rates of dental abnormalities. Therefore, we evaluated whether peroral endoscopy can facilitate the detection of dental abnormalities. In this study, we explored the feasibility of utilizing peroral endoscopy as a novel approach for detecting dental abnormalities.

## Materials and methods

Study design and patients

This was a retrospective study at the Okayama Rosai Hospital, Okayama, Japan. Patients who underwent peroral endoscopy between July and September 2022 were retrospectively evaluated. Participants in whom the oral cavity was captured during the endoscopy procedure were included in the study. Patients in whom no teeth were visible on the photographs were excluded. The study design was approved by the Ethics Committee of Okayama Rosai Hospital (approval number: 460) and complied with the Declaration of Helsinki.

The included cases were selected by Ryo Kato, a Board Certified Fellow of the Japan Gastroenterological Endoscopy Society, who independently reviewed all images obtained from upper gastrointestinal endoscopy examinations conducted during the study period. If some teeth were visible, the location of the teeth, presence or absence of any abnormalities, and abnormalities, if any, were recorded. Patient sex and age at the time of examination were retrospectively reviewed from the clinical records.

Endoscopes and techniques of observation of teeth

Four types of upper gastrointestinal endoscopes (GIF-Q260J, GIF-H290, GIF-H290Z, and GIF-XZ1200; Olympus Medical Systems, Tokyo, Japan) were randomly used in this study. Intraoral images were obtained when the endoscope was inserted into the patient’s mouth while wearing a mouthpiece. There was no standardized protocol for oral observation at the facility; both the method of capturing intraoral images and the decision of whether to capture them were left to the discretion of the endoscopist.

Endpoint

**Figure 1:**
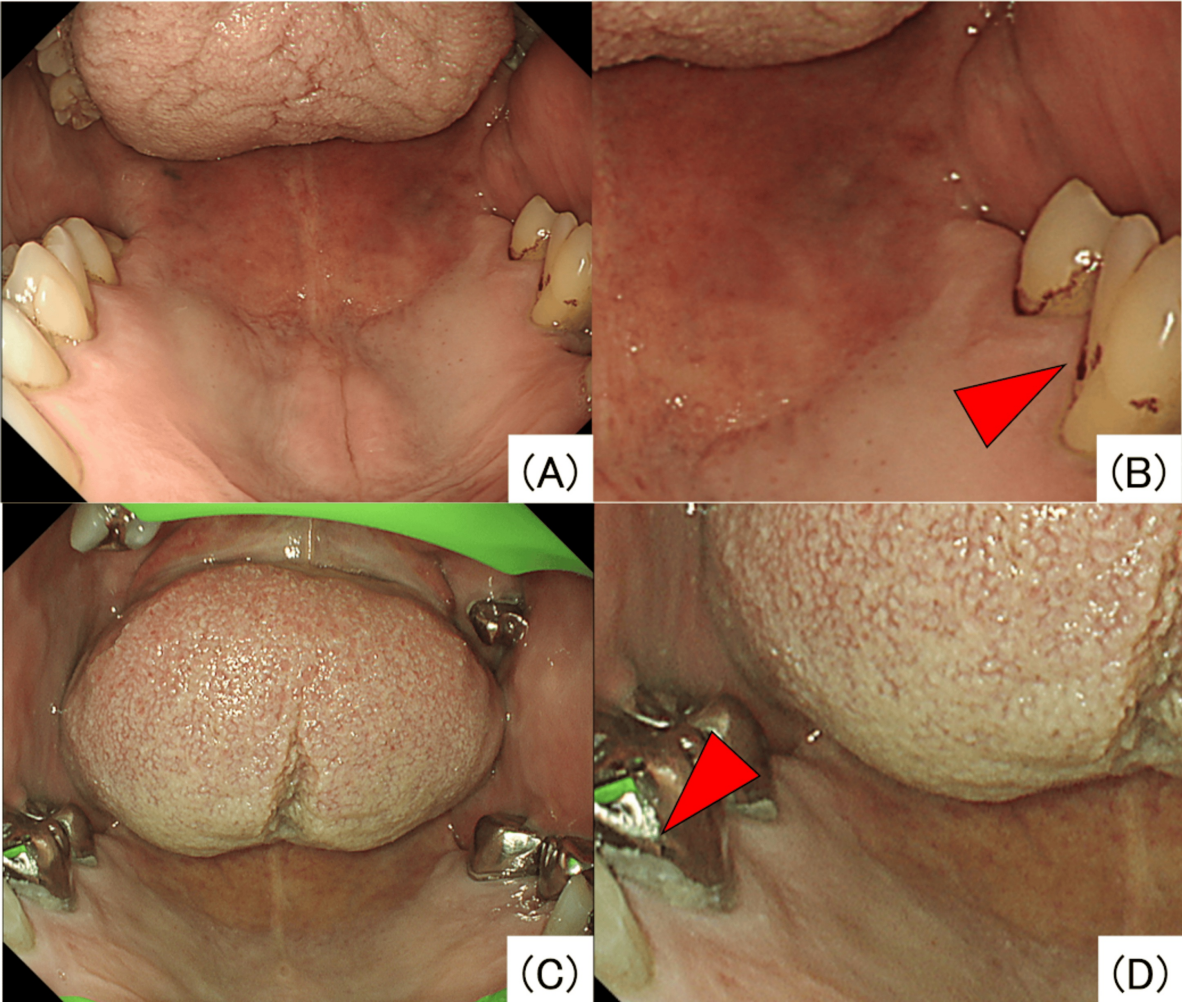
Endoscopic images showing pigmentation (A, B) and residual plaque (C, D).

**Figure 2:**
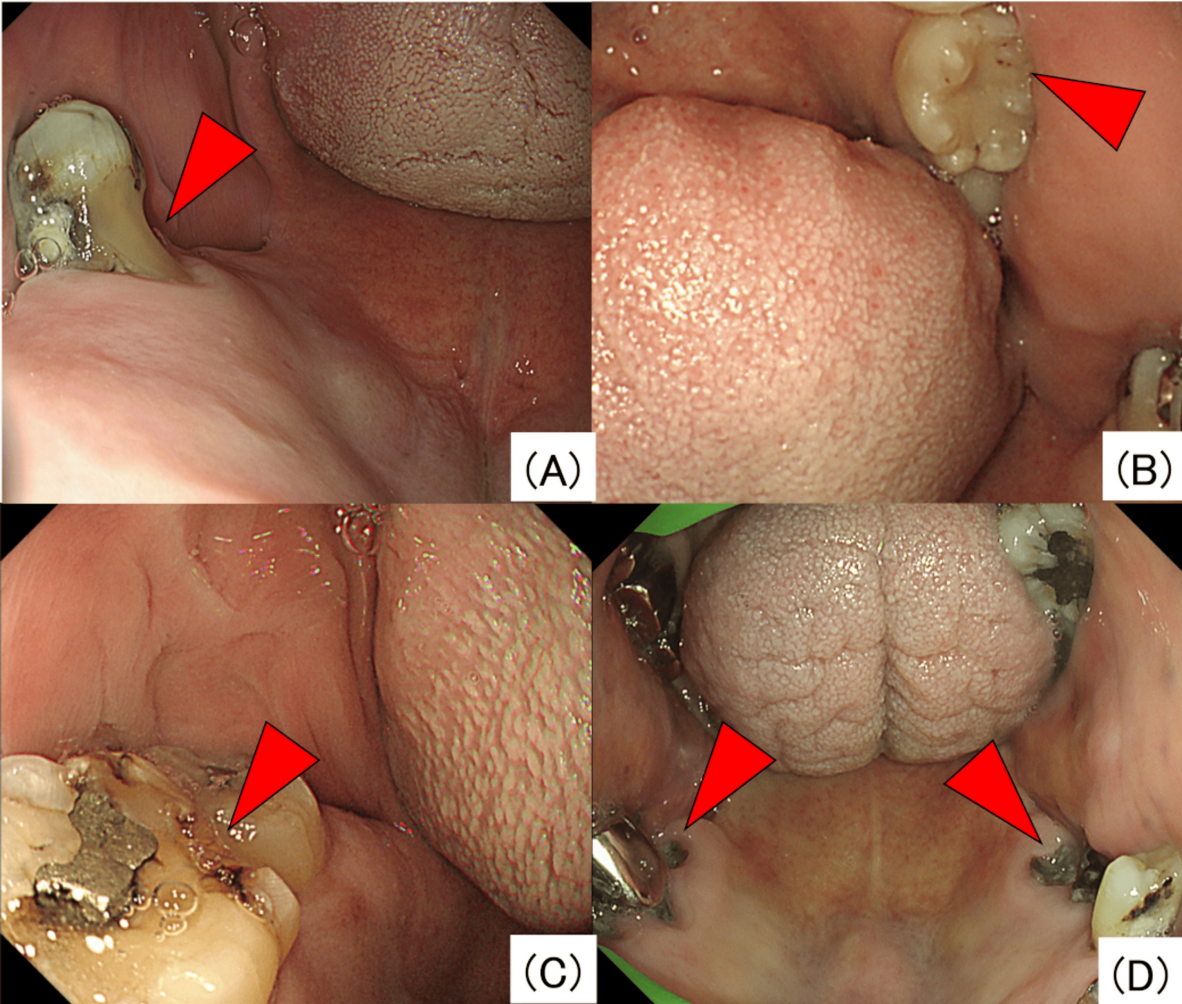
Endoscopic images showing roots exposure (A), black spots suspected of caries (B), defects (C), and residual roots (D).

The primary endpoint was the frequency of dental abnormality detection. In this study, the following six findings were defined as dental abnormalities: pigmentation (Figures 1A, 1B), residual plaque (Figures 1C, 1D), root exposure (Figure 2A), black spots suspected of caries (Figure 2B), defects (Figure 2C), and residual roots (Figure 2D). These abnormalities in coloration and morphology were selected as findings that even endoscopists, who were not dental specialists, could readily notice.

## Results

During the study period, 918 upper gastrointestinal endoscopies were performed. The oral cavities were examined in 104 patients. No teeth were visible in 32 patients; consequently, 72 patients (43 men, 29 women, median age 70 (range 42-88) years) were evaluated for dental abnormalities. Figure 1 shows the flowchart for participant selection.

**Figure 3:**
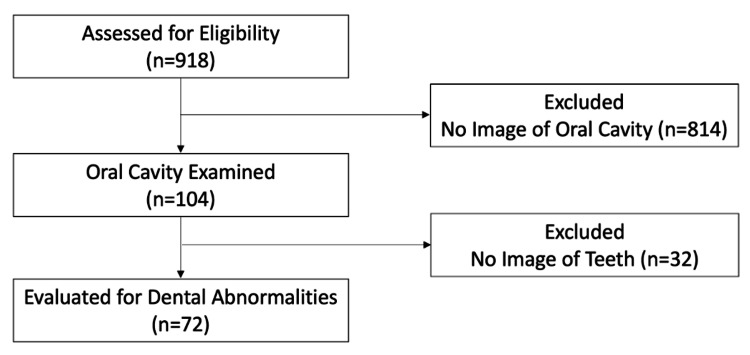
Flow diagram showing participant selection

All the 72 endoscopic examinations were conducted for the purpose of health checkups or routine screenings. There was a substantial amount of missing information on the patients' underlying conditions, medical conditions, drinking, smoking, and frequency of dental checkups, which limited the ability to evaluate potential risk factors associated with dental abnormalities.

Frequency of detection of dental abnormalities

All the captured teeth were molars. Of the 72 patients assessed, dental abnormalities were detected in 28 (39%; 95%CI, 27.7%-50.3%). The most common abnormality was residual plaque (n=10), followed by pigmentation (n=7), root exposure (n=5), black spots suspected of caries (n=4), defects (n=1), and residual roots (n=1).

**Table 1:** Details of 28 dental abnormalities

Pigmentation	Residual plaque	Root exposure	Black spots suspected of caries	Defects	Residual roots
n=7	n=10	n=5	n=4	n=1	n=1

## Discussion

We found a high frequency of dental abnormalities (39%; 95%CI, 27.7%-50.3%); 28 of 72) in patients whose oral cavity was imaged at the time of peroral endoscope insertion, although the study was conducted over a limited period and in a limited number of patients.

Pigmentation is not morbidly significant, as it is mostly caused by consumption of polyphenol-rich foods, such as coffee, red wine, and chocolate, as well as by smoking. In contrast, other findings were considered abnormalities requiring oral care instruction and dental treatment. Residual plaque can cause periodontal disease and dental caries. Root exposure is caused by gingival recession due to periodontal disease, which increases the risk of developing dental caries. Black spots, defects, and residual roots were considered findings indicating caries.

Many periodontal disease-related abnormalities were detected in this study. In Japan, more than 50% of elderly individuals have periodontal disease [7]. Periodontal disease is a cause of aspiration pneumonia, which is a major cause of death among the elderly, and has also been implicated in diabetes mellitus and myocardial infarction [12-17]. While theories vary, periodontal disease is thought to influence increased insulin resistance and the progression of atherosclerosis via elevated tumor necrosis factor-alpha (TNF-α) production [18]. Furthermore, it has been suggested that oral bacteria associated with periodontal disease may directly enter the bloodstream and cause inflammation in the vascular walls [19]. Moreover, periodontal disease is the most common reason for tooth extraction. Detection of periodontal disease-related abnormalities using peroral endoscopy may lead to therapeutic intervention for these diseases.

It is also significant that many caries-related abnormalities were detected in this study. Black spots may represent an early stage of caries. Therefore, dental examinations are desirable to prevent the progression of caries. Tooth defects and residual roots are advanced caries-related conditions. When the dental nerve is damaged due to caries progression, subjective symptoms sometimes disappear, and the caries may be left untreated. However, there is a concern that it may progress to osteomyelitis and adversely affect the surrounding teeth; therefore, immediate treatment is necessary.

The teeth can be observed in an extremely short time (a few seconds) before the scope is inserted into the pharynx. Moreover, especially in regions where endoscopic screening is widespread, this method does not require additional equipment or medication, making it cost-effective and immediately applicable in clinical practice. Peroral endoscopy may expand the opportunities for detecting dental abnormalities with minimal effort.

This study has some limitations. First, the sample size is small. However, this was an exploratory pilot study, and this limitation should be addressed in future studies. Second, there may have been a selection bias. This retrospective study included individuals whose oral images were captured, although images of the oral cavity may not have been routinely captured during peroral endoscopy. Therefore, the actual prevalence of dental abnormalities may be lower than that observed in this study. Third, we did not evaluate whether endoscopic detection of dental abnormalities led to dental examinations or treatment. There could be a possibility that cases not actually requiring dental examinations or intervention may also be identified. Fourth, due to a substantial amount of missing data on patients’ underlying conditions and other factors, we were unable to analyze risk factors for dental abnormalities. Fifth, in this study, endoscope models were randomly selected, and variations in image quality may have occurred among different models. However, since all endoscopes had sufficient resolution to detect early gastric cancer, the impact on the accuracy of dental abnormality detection was considered negligible. Finally, only molars were observed in this study because of the mouthpiece. However, molars are common sites of caries [6], and observing them during peroral endoscopy is recommended when screening for dental abnormalities.

## Conclusions

Detection of dental abnormalities during peroral endoscopy is feasible, and this new approach could potentially offer opportunities for the detection of dental abnormalities in patients undergoing peroral endoscopy. As this was a retrospective pilot study, a prospective study is warranted to evaluate the utility of this method. In such a study, establishing a referral protocol for patients with endoscopic dental abnormalities to receive dental evaluation would support timely interventions and help validate endoscopic findings. Furthermore, collecting patient data, including underlying medical conditions, drinking and smoking history, and dental checkup frequency, may help identify risk factors associated with dental abnormalities. And, by establishing standardized oral imaging protocols and unifying the endoscope models used during prospective studies, investigations could be conducted with greater reproducibility.

## References

[1] Yamamoto T, Kondo K, Hirai H, Nakade M, Aida J, Hirata Y (2012). Association between self-reported dental health status and onset of dementia: a 4-year prospective cohort study of older Japanese adults from the Aichi Gerontological Evaluation Study (AGES) project. Psychosom Med.

[2] Yamamoto T, Kondo K, Misawa J (2012). Dental status and incident falls among older Japanese: a prospective cohort study. BMJ Open.

[3] Aida J, Kondo K, Hirai H (2012). Association between dental status and incident disability in an older Japanese population. J Am Geriatr Soc.

[4] Suzuki S, Sugihara N, Kamijo H (2022). Reasons for tooth extractions in Japan: the second nationwide survey. Int Dent J.

[5] The Ministry of Health, Labor and Welfare of Japan (2017). The Ministry of Health, Labor and Welfare of Japan: Dental disease survey [Website in Japanese]. https://www.mhlw.go.jp/toukei/list/62-17.html.

[6] Gilbert GH, Foerster U, Dolan TA, Duncan RP, Ringelberg ML (2000). Twenty-four month coronal caries incidence: the role of dental care and race. Caries Res.

[7] Aida J, Ando Y, Akhter R, Aoyama H, Masui M, Morita M (2006). Reasons for permanent tooth extractions in Japan. J Epidemiol.

[8] Statistics Sweden (2024). Statistics Sweden: Statistics on dental health. https://www.socialstyrelsen.se/publikationer/statistics-on-dental-health-2024-2025-5-9599/.

[9] Muto M, Minashi K, Yano T (2010). Early detection of superficial squamous cell carcinoma in the head and neck region and esophagus by narrow band imaging: a multicenter randomized controlled trial. J Clin Oncol.

[10] Hamada K, Ishihara R, Yamasaki Y (2018). Transoral endoscopic examination of head and neck region. Dig Endosc.

[11] Iwamuro M, Hamada K, Kawano S, Kawahara Y, Otsuka M (2023). Review of oral and pharyngolaryngeal benign lesions detected during esophagogastroduodenoscopy. World J Gastrointest Endosc.

[12] Khadka S, Khan S, King A, Goldberg LR, Crocombe L, Bettiol S (2021). Poor oral hygiene, oral microorganisms and aspiration pneumonia risk in older people in residential aged care: a systematic review. Age Ageing.

[13] Borgnakke WS, Ylöstalo PV, Taylor GW, Genco RJ (2013). Effect of periodontal disease on diabetes: systematic review of epidemiologic observational evidence. J Clin Periodontol.

[14] Larvin H, Kang J, Aggarwal VR, Pavitt S, Wu J (2021). Risk of incident cardiovascular disease in people with periodontal disease: a systematic review and meta-analysis. Clin Exp Dent Res.

[15] Okuda K, Kimizuka R, Abe S, Kato T, Ishihara K (2005). Involvement of periodontopathic anaerobes in aspiration pneumonia. J Periodontol.

[16] Grossi SG, Genco RJ (1998). Periodontal disease and diabetes mellitus: a two-way relationship. Ann Periodontol.

[17] Bahekar AA, Singh S, Saha S, Molnar J, Arora R (2007). The prevalence and incidence of coronary heart disease is significantly increased in periodontitis: a meta-analysis. Am Heart J.

[18] Katagiri S, Nitta H, Nagasawa T (2009). Multi-center intervention study on glycohemoglobin (HbA1c) and serum, high-sensitivity CRP (hs-CRP) after local anti-infectious periodontal treatment in type 2 diabetic patients with periodontal disease. Diabetes Res Clin Pract.

[19] Yamazaki K, Ohsawa Y, Itoh H (2004). T-cell clonality to Porphyromonas gingivalis and human heat shock protein 60s in patients with atherosclerosis and periodontitis. Oral Microbiol Immunol.

